# “Teachers' Emotional Labor” Publications in Web of Science: A Bibliometric Analysis

**DOI:** 10.3389/fpsyg.2022.899261

**Published:** 2022-05-18

**Authors:** Aihui Wu, Rining Wei

**Affiliations:** ^1^College English Teaching Department, Jiangsu University, Zhenjiang, China; ^2^Department of Applied Linguistics, Xi'an Jiaotong-Liverpool University, Suzhou, China

**Keywords:** Web of Science, teachers' emotional labor, bibliometric characteristics, CiteSpace, synthesis

## Abstract

One of the indicators that symbolize the success of an academic field is its academic publications in well-established citation indices. This article first explored the bibliometric characteristics of publications on “teachers' emotional labor” (TEL) in the Social Sciences Citation Index (SSCI) and the Arts & Humanities Citation Index (A&HCI), two prestigious citation indices available in the Web of Science (WoS). Search with the term “teacher emotional labor” retrieved 173 publications that included this term in their titles, abstracts, or keywords in the WoS database between 1900 and 2020. The bibliometric characteristics pertaining to numbers of publications, document types, research categories, research areas, authors, journals, universities, and countries were reported. Then, CiteSpace was utilized to visualize TEL research and to obtain insights into its research focuses and its future directions. The findings will contribute to TEL research by informing scholars in the fields of L2 research and psychology and others.

## Introduction

Emotions are inherently interwoven in teaching for teaching is a human-service profession and teachers are engaged in interpersonal interactions. They juggle multiple commitments and cater to large numbers of students in particular, on a daily basis and for an extended period of time. Teaching has thus been described as a profession characteristic of a high-wire act (Winograd, 2005) because teachers often struggle between their felt emotions and required emotions. Good teachers are supposed to be in a constant display of positive emotions (Hargreaves, 1998), attend to the emotional needs of learners, and manage their emotional states as expected (Isenbarger and Zembylas, 2006). Their emotions are contagious and have an impact on students' learning attitude and motivation (Moskowitz and Dewaele, 2021) and academic achievements (Burić and Frenzel, 2020). A large body of research, however, has found that the “management of feeling to create a publicly observable facial and bodily display,” which is referred to as emotional labor (EL) (Hochschild, 1983, p. 7), can impact teachers' instructional behavior and can lead to burnout in the long run (*cf*. Yin et al., 2019). The very negative associations with EL have attracted growing attention from researchers across disciplines, ranging from sociology to management science to psychology.

The rapid expansion and development of EL research brings about some inconsistency in EL interpretation. Scholars in sociology tend to utilize occupational requirements, those in organizational behavior tend to rely on the conceptualization of emotional displays, whereas those in psychology tend to draw upon the intrapsychic process (*cf*. Grandey et al., 2013). These separate lenses make the understanding of EL void of transparency, which thus points to a need to embrace a more encompassing perspective in investigating EL (see Grandey et al., 2013; Dewaele and Wu, 2021). For EL research in the educational context, scholars have drawn inspiration from these lenses. Coupled with the uniqueness of “personal and work characteristics” (Grandey and Melloy, 2017, p. 409) of teaching, a plethora of EL research works have been brought forth. Particularly, with the advent of the “affective turn” in the social science and humanities, Prior (2019, p. 518) highlighted the significant relevance of language to emotions. Accordingly, language teachers' EL has garnered more attention compared with other subject teachers' EL, for “language classrooms are inherently stressful environments for some people” (Gkonou et al., 2020, p. 1). Despite this claim, there still exists a “significant lack of research in applied linguistics on the roles of teacher emotion labor and emotional intelligence in FL classrooms” (Acheson et al., 2016, p. 525).

However, very few studies (e.g., Yin et al., 2019, refer to the “Literature review” section) have attempted to synthesize the research on teachers' emotional labor (TEL). Besides, to the best of our knowledge, no attempts have been made to review the research in this area by utilizing bibliometrics, which is a mathematics and statistical method widely employed to provide quantitative research assessment of academic publications (Pritchard, 1969). This article is intended to fill this gap by making use of a bibliometric analysis combined with CiteSpace to present a quantitative and visualized description of current academic publications on TEL. Specifically, in the remainder of this study, following the relevant literature, the study design combining bibliometrics and visualized results from CiteSpace is explained, and then the major results and the discussion are presented. The conclusion presents some implications and suggestions for future research in the field of TEL.

## Literature Review

Emotions are at the heart of teaching (Hargreaves, 1998). The labor that teachers invest in managing emotions for better interpersonal interactions was identified by the prominent American sociologist Hochschild (1983) as EL nearly three decades ago. This term emerged as a response to the rapid rise of service sectors where Hochschild observed that the flight attendants' labor in managing their feelings (i.e., putting up phony smiles) is analogous to the physical labor of the wallpaper factory child in that both are aimed for gaining wages in return. The “affective turn” in organizational behavior (Barsade et al., 2003, p. 3) and later in education further fueled the research on TEL, resulting in the “lift-off of emotional research in applied linguistics” since 2010 (Dewaele, 2019, p. 533). Generally, two research camps influencing current TEL research have emerged in the past decade: what EL is (Grandey et al., 2013) and what EL can do socially (Benesch, 2017; Gkonou and Miller, 2021), or “structure research” vs. “poststructure research” (Benesch, 2017, p. 34). The former approach deconstructs EL into several components (i.e., surface acting) and looks into and measures how they are correlated to other features (i.e., emotional intelligence, see Dewaele and Wu, 2021), while the latter looks at “what emotions do socially, not what they are inside people's minds” (Benesch, 2017, p. 7). We take this divide to review the studies that are relevant to this study.

To reach an agreement on the conceptualization of EL, *viz*. what EL is, has always guided the EL research in various disciplines to date. How emotions are managed or what strategies can be adopted to regulate emotions in the interaction (Grandey, 2000; Dewaele and Wu, 2021) is the key component of EL. Hochschild (1983) proposed surface acting (faking expressions or suppressing emotions) and deep acting (conjuring up desired feelings). Building upon these two strategies, other researchers have managed to enrich them. Diefendorff et al. (2005) added a strategy of expressions of naturally felt emotion, and Yin (2012) validated the three-dimensional Teacher Emotional Labor Strategy Scale (TELS) for Chinese primary and secondary teachers. In contrast, Çukur (2009) developed another TELS with four dimensions, namely, surface acting, deep acting, automatic emotion regulation, and emotional deviance among Turkish high school teachers. Interestingly, Zhang et al. (2020a) also claimed to validate four dimensions for emotion regulation among Chinese preschool teachers, but their dimensions (i.e., disguising, restraining, self-persuading, and releasing) differ significantly from those in Çukur (2009). The inconsistency of the instruments for measuring TEL attests to the complexity of the construct and the multiplicity of its theoretical lens and points to a need for further research.

Grandey (2000) drew upon the previous EL literature and proposed a conceptual model of EL, which viewed EL as emotion regulation. She contended that the mechanisms of EL lie in “emotion regulation” (Gross, 1998) where employees regulate their physiological arousal and cognitions to display the desired feelings (also see Dewaele and Wu, 2021). In her tripartite model, Grandey (2000) included three antecedents [i.e., individual factors, organizational factors, and situational cues (i.e., situational expectations and emotional events)], the managing/regulating process (i.e., surface acting and deep acting), and two consequences (i.e., individual wellbeing and organizational wellbeing).

Largely drawing on Grandey (2000) EL model, TEL research reviews to date have demonstrated similar insights into the focuses and findings. For example, Wang et al. (2019) conducted a summative literature review supplemented by a meta-analysis based on Grandey's EL model, and they added a managing process (i.e., genuine expression) and slightly changed TEL consequences (i.e., psychological wellbeing, physical health, and behavioral engagement). Based on 28 empirical articles from three databases [i.e., ERIC, PsycINFO, and Web of Science (WoS)], they found that surface acting is detrimental to teachers' psychological wellbeing, while genuine expression of emotions is beneficial. However, the effects of deep acting are culturally contradicted (i.e., eastern vs. western).

Likewise, Yin et al.'s (2019) conducted a meta-analysis with an expanded dataset (85 empirical articles from six databases between 2000 and 2017 and additional search results through Google Scholar). Their analysis model included the antecedents of job characteristics (e.g., job demands, job autonomacy/control, and social support) and individual characteristics (e.g., emotional intelligence) and the consequences of burnout (e.g., emotional exhaustion, depersonalization, and reduced accomplishment/efficacy) and job satisfaction. Their study had similar findings regarding the consequences in view of the relationships between the three components of EL and burnout and job satisfaction. Moreover, the antecedents in both review articles are complicated and various, roughly divided between individual factors and contextual factors, indicating a need for more systematic investigation into this aspect.

However, beyond the camp of structure research evidenced by meta-analytical reviews above, the emotional realities of teaching are complex and nuanced due to the “discipline-specific challenges” teachers have to face (Loh and Liew, 2016, p. 270). This situation has prompted the other camp of researchers to explore what EL does socially in different subject contexts. In addition to a few earlier research works on science teachers' EL (i.e., Zembylas, 2003), more recent scholars have sought to examine language teachers' (predominantly EFL teachers') EL in view of the uniqueness of being highly culturally responsive in language teaching (Loh and Liew, 2016). This camp of EL research is typically characterized by a sociopolitical lens or a poststructuralist approach (Benesch, 2017; Gkonou and Miller, 2021), as evidenced by Benesch's (2017, p. 6) claim that EL is the “result of interaction between different spheres of power, including institutional, professional, and individual.” She suggested that language teachers' EL is more likely to be generated by the stickiest teaching issues [i.e., high-takes literacy testing, also see Loh and Liew (2016)]. Similarly, King's (2016, p. 109) study on five mid- to late-career expatriate EFL teachers in a Japanese university revealed that language teachers' EL could be added by “extensive reforms to curriculum content, course structure, and the administrative duties.” Conversely, Gkonou and Miller's (2021) interviews with 25 high school language teachers from the United States and the United Kingdom revealed that EL can be beneficial in helping build up their emotional capital, which is then converted into social and cultural capital.

Nonetheless, the above reviews have revealed some hot issues in EL research in connection with this study. Yet, the extant reviews and meta-analytical studies have their limitations; for instance, one inherent limitation of a meta-analysis is that its scope has to be confined to (primarily) quantitative studies that can generate effect sizes (Wei et al., 2019). More review studies employing a bibliometric method such as this study will contribute to the understanding of the disciplinary trends that researchers in a particular field are interested in and allow them to reflect on the developments of this field (Arik and Arik, 2015), as bibliometric analyses can include both quantitative studies and their counterparts with vivid visualization. This study thus aims to scrutinize how teachers' EL research has been progressing both quantitatively and visually and to signpost directions for future research.

## The Present Study

### Databases, Research Questions, and Analytic Strategy

This study aims to contribute to TEL research by providing a quantitative and visualized analysis through bibliometrics and CiteSpace. Thomson Reuters' WoS was chosen as the database for analysis for three major reasons. First, WoS has been commonly chosen as the data source for bibliometric analyses studies in recent years (e.g., Cansun and Arik, 2018; Guo et al., 2020; Karakus et al., 2021). Second, WoS is one of the most established databases, which were built in 1900 with SSCI launched in the same year and A&HCI in 1975. Therefore, SSCI and A&HCI were selected for full coverage of files relating to the term “emotional labor” coined by Hochschild (1983: ix). Third, WoS provides a large database across 256 disciplines for people to search and analyze and generates more accurate information about the publications compared with other databases (Mongeon and Paul-Hus, 2015; Cansun and Arik, 2018).

To generate the database for the bibliometric and the visualized analyses, two major steps were conducted. Step one involved a search in the databases of SSCI and A&HCI in the WoS Core Collection. Under the basic search mode, the term “teachers' emotional labor” was entered in the “Topic” search field, with the timespan set between 1900 and 2020. To be specific, to search in the “Topic” field in WoS means searching the entered term in the fields of Title, Abstract, Author Keywords, and Keywords Plus. A total of 214 publications were retrieved as a result. Step two entailed creating the exclusion criteria to refine the results (*cf*. Karakus et al., 2021). The two authors carefully read the information of each study (i.e., the abstract and its bibliometric characteristics) and particularly examined its relevancy to TEL research. Three key exclusion criteria were thus agreed on. (1) Studies without teachers as the participants were excluded. (2) Studies with irrelevant contents were excluded. (3) Studies with abstracts only were excluded. With these filtering methods, 173 publications out of 214 were finally retrieved to form the core dataset, which was also loaded into CiteSpace to visualize the data after their bibliometric characteristics were reported. With this research design in mind, this study attempts to address the following research questions:

RQ1. What are the bibliometric characteristics (e.g., document types) of the publications?
RQ2. What is the focus of the publications?

RQ1 was addressed with a quantitative analysis *via* WoS, featuring bibliometric characteristics including numbers of publications, document types, research categories, research areas, authors, journals, author affiliations, and countries. For RQ2, it was answered by presenting visualizations *via* CiteSpace and the concomitant synthesis.

## Results and Discussion

### The Bibliometric Characteristics

#### Numbers of Publications

**Figure 1:**
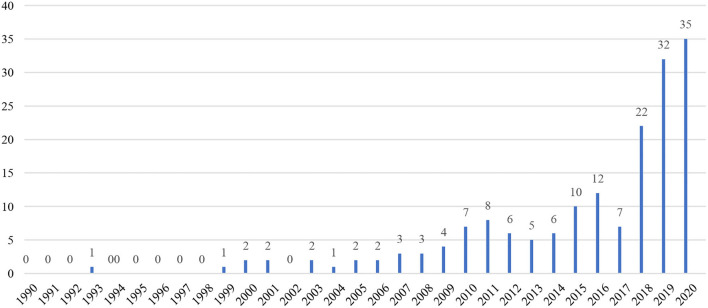
The number of teachers' emotional labor (TEL) publications in the Web of Science (WoS) per year.

Figure 1 shows the yearly distribution of publications on TEL between 1990 and 2020. Among the 173 publications, the first one appeared in 1993, 10 years after the term “emotional labor” appeared. From 2000 to 2010, TEL output remained constant between two and four each year. Due to a “lift-off of emotion research” in applied linguistics around the turn of the millennium (Dewaele, 2019), TEL research between 2010 and 2017 began to gain momentum with a slight jump from three on a yearly basis to eight publications per year. A spike of 22 publications occurred in 2018, doubling the yearly output in previous years. Recently, the output has been over thirty each year, with a peak of 35 publications in 2020. It can be predicted that in the years to come, research on TEL can be more productive.

#### Document Types

Of the 173 publications in Table 1, a total of 163 publications were regular journal articles. Among them, five were book reviews, four were editorial materials, and one was proceedings paper. It is obvious that publications were predominantly published in the form of articles.

**Table 1:** Document types for teachers' emotional labor (TEL) publications in Web of Science (WoS).

**Document types**	**Count**	**Percentage (out of 173)**
Article	163	94.22
Review	5	4.62
Editorial material	4	3.47
Proceedings paper	1	0.58

#### Research Categories

According to Table 2, education/educational research had the most publications (96, 55.49%), followed by psychology multidisciplinary (19, 10.98%). The total number of the second column in Table 2 surpassed 173, indicating certain overlaps among the categories defined by WoS.

**Table 2:** Distribution of TEL publications according to WoS categories.

**WoS category**	**No. of publications**	**Percentage (out of 173)**
Education/Educational research	96	55.49
Psychology multidisciplinary	19	10.98
Psychology educational	16	9.25
Psychology applied	14	8.10
Public environmental/occupational health	13	7.51
Linguistics	10	5.78
Environmental sciences	6	3.47
Sociology	6	3.47
Language Linguistics	5	2.89
Psychology	5	2.89

#### Research Areas

In Table 3, education/educational research had the most TEL publications (97, 56.1%), followed by psychology (56, 32.37%). In contrast, other areas with publications over ten are public environmental occupational health (13, 7.50%) and linguistics (10, 5.78%). It is worth noticing that publications in the area of linguistics were ranked fourth in the list, which could be attributed to recent increasing attention to language teachers' TEL research.

**Table 3:** Distribution of TEL publications per WoS research area.

**WoS research area**	**No. of publications**	**Percentage**
		**(out of 173)**
Education/Educational research	97	56.10
Psychology	56	32.37
Public environmental occupational health	13	7.50
Linguistics	10	5.78
Environmental sciences ecology	8	4.62
Business economics	6	3.47
Sociology	6	3.47
Psychiatry	5	2.89

#### Authors

In Table 4, H. Yin was the most productive author, contributing up to 13 TEL publications. A. Frenzel and S. Huang had six publications, while the other three authors had five. A total of 338 authors have contributed 173 TEL publications in WoS, with an average of 1.95 authors per publication.

**Table 4:** The authors contributing most to TEL publications.

**Authors**	**No. of publications**	**Percentage**	**Document types**
		**(out of 173)**	
H. Yin	13	7.51	Article (12), Review (1)
A. Frenzel	6	3.47	Article (6)
S. Huang	6	3.47	Article (5), Review (1)
I. Buric	5	2.89	Article (5)
J. Taxer	5	2.89	Article (4), Review (1)
M. Zembylas	5	2.89	Article (3), Review (2)

#### Journals

Table 5 shows that *Teaching and Teacher Education* published the most TEL documents (18, 10.40%), followed by *Frontiers in Psychology* (12, 6.94%). The latter is an open access journal, which means that it may attract more attention in the long run and hence have more (if not most) TEL publications in the future.

**Table 5:** Journals with the most TEL publications in WoS.

**Journal**	**Count**	**Percentage (out of 173)**
Teaching and Teacher Education	18	10.40
Frontiers in Psychology	12	6.94
Teachers College Record	7	4.05
Educational Psychology	5	2.89
Teacher and Teaching	5	2.89

#### Author Affiliations

In terms of author affiliations, the Chinese University of Hong Kong contributed most TEL publications (15, 8.67%), followed by the University of Munich (6, 3.47%) and Shanghai Normal University (5, 2.89%). Among these top three universities with over five publications, two of them are from China, suggesting that China-based scholars are making a major contribution to TEL research across the world.

#### Countries

Out of the 11 countries mentioned in Table 6, the United States ranked first (44, 25.43%), followed by the People's Republic of China (37, 21.39%). These two countries combined have made up nearly half of TEL publications.

**Table 6:** Top nine countries with TEL publications.

**Country**	**Count**	**Percentage (out of 173)**
USA	44	25.43
P. R. China	37	21.39
England	20	11.56
Germany	13	7.51
Australia	12	6.94
Turkey	9	5.20
Canada	8	4.62
Netherlands	7	4.05
Croatia	6	3.47
Israel	6	3.47
Spain	6	3.47

The bibliometric analysis above reflects the general features of TEL publications from 1993 (with the first TEL publication) to 2020. A total of 214 publications were retrieved from the databases of SSCI and A&HCI in the WoS Core Collection, and 173 publications were used for analysis after refinement. The number of annual publications peaked in 2020 (*n* = 35). The types of publications were predominantly journal articles (*n* = 163). The research category and the research area are another two bibliometric indicators of the TEL publications. They both had the most TEL publications (*n* = 96 and *n* = 97, respectively) in education/educational research. The top two journals abundant in TEL research were *Teaching and Teacher Education* (*n* = 18) and *Frontiers in Psychology* (*n* = 12). The most productive author and the affiliation of the authors were both based in China (H. Yin, *n* = 13; the Chinese University of Hong Kong, *n* = 15), while the country highest in TEL research output was the United States (*n* = 44). The information above has provided us with quantitative descriptions of TEL research in the past three decades. These descriptions can be usefully complemented with the qualitative knowledge based on the visualized analysis of co-citations *via* CiteSpace.

### The Focus

#### Co-citation

The co-citation analysis is a quantitative method used to map and visualize the structure and dynamics of specialties. It is often employed by researchers to help obtain insights into emergent patterns (Chen et al., 2010). Small and Griffith (1974) proposed the approach of studying a network of co-cited references to explore the content of a knowledge domain by cluster analysis of document co-citation network (DCA), which is based on the fact that when two individual items are co-cited, these two items are more likely to be semantically related (Guo et al., 2020). According to Small (1978), cited documents (e.g., references) are concept symbols for scientific ideas, methods, and experiments; therefore, co-citation clusters can reveal underlying intellectual structures (Chen et al., 2010).

The following section presents a visualized analysis of co-citations by means of CiteSpace. A total of 173 documents with distinct 7,304 references were loaded into CiteSpace (version: 5. 7. R2) to form the reference co-citation clusters. In line with the common practice, the network was constructed under the selection criteria that the top 10% of the most cited or occurred items were kept in each slice with the maximum number of selected items of no more than 100 per slice. After pilot testing, the threshold level for citation count was set at eight documents to allow more clusters to emerge, and the CiteSpace configuration was constructed with link retaining factor = 3, look back years = 5, e = 1.0, and g-index (*k* = 80).

**Figure 2:**
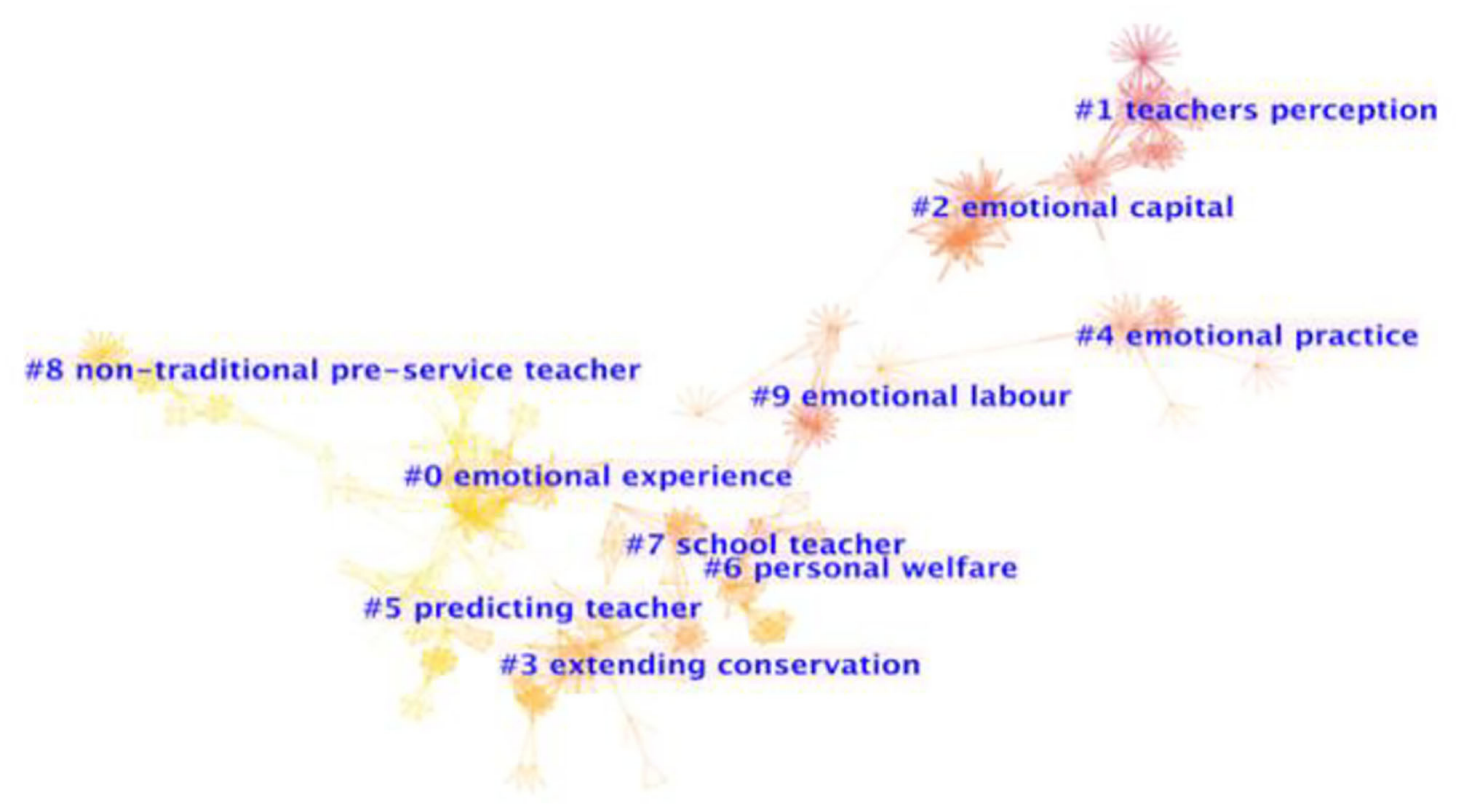
Cluster view of reference co-citation network in TEL documents during 1993–2020.

The final co-citation network was formed with 1,323 merged nodes and 4,542 edges, resulting in ten major co-citation clusters with more than 38 documents in each cluster to form the main cluster view (Figure 2) aligned with the common practice in other studies (i.e., Guo et al., 2020). Modularity and silhouette are two indicators used to assess how well the field is split into clusters (*cf*. Zhang et al., 2020b). The resulting modularity (Q) value is close to 1 (*Q* = 0.93, 0 < Q <1), indicating dense connections between the nodes, and a high valued weighted mean silhouette (S) (S = 0.97, −1 < S < 1) means a good match between the object and its own cluster.

#### The Focus

**Figure 3:**
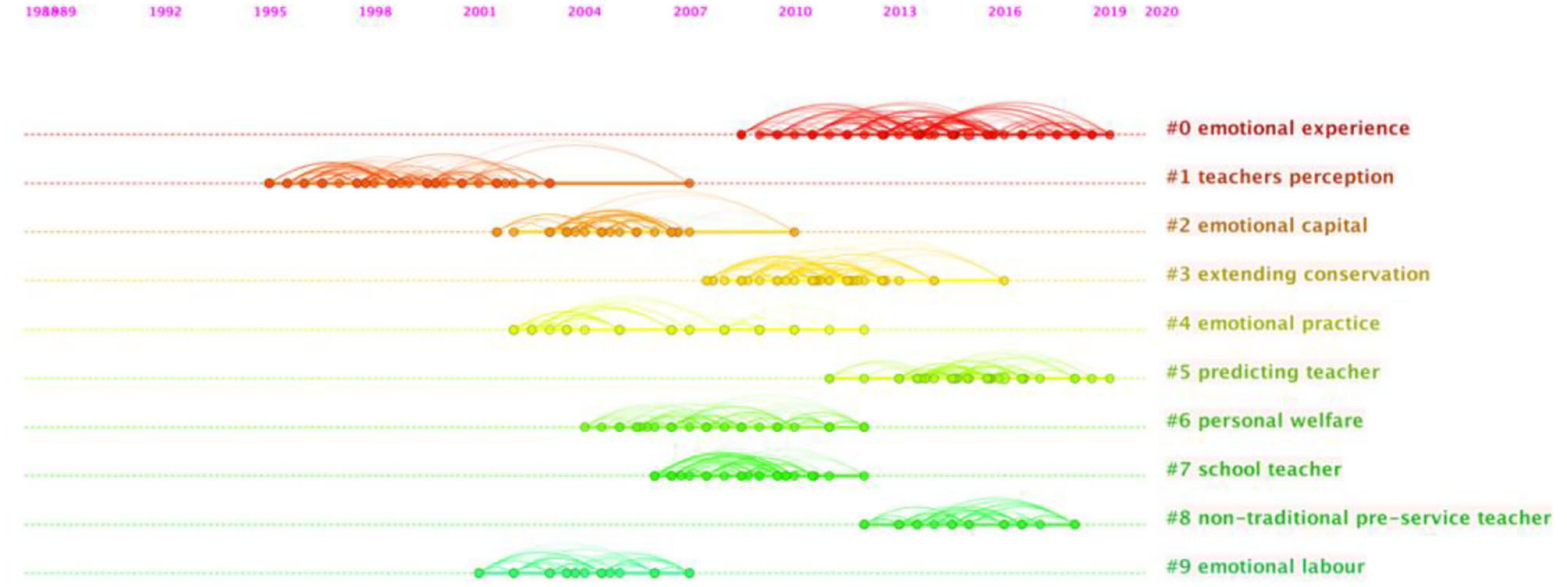
A timeline view of reference co-citation network in TEL documents during 1993–2020.

Table 7 reports the detailed information of the ten largest clusters, whose labels were selected by their citers based on the tf^*^idf term ranking algorithm, which tends to represent the most salient aspect of a cluster (Chen et al., 2010). Besides, the silhouette values of the ten major clusters presented in Table 7 were mostly close to 1, indicating that the clustering configurations are appropriate. For a clearer understanding of the evolution footprints of TEL research over the past years, a timeline view of the clusters was exported (Figure 3).

**Table 7:** Specific information on the 10 major clusters.

**Cluster ID**	**Cluster label**	**Silhouette**	**Size**	**Mean** **(citee year)**	**Representative documents**
#0	Emotional experience	0.954	110	2013	Keller et al., 2014; Donker et al., 2020; Liao et al., 2020
#1	Teachers perception	0.973	96	1998	Hargreaves, 2000; Winograd, 2003; Brown et al., 2018
#2	Emotional capital	0.959	73	2004	Zembylas, 2003, 2007; Yin, 2012
#3	Extending conservation	0.958	73	2010	Park et al., 2014; Yin, 2015; Lee and Van Vlack, 2018
#4	Emotional practice	1	70	2005	Jenkins and Conley, 2007; Steinberg, 2008; Mackenzie, 2012
#5	Predicting teacher	0.863	60	2015	Özdemir and Koçak, 2018; Huang et al., 2020; Kang, 2020
#6	Personal welfare	1	52	2008	Purvanova and Muros, 2010; Barber et al., 2011; Basim et al., 2013
#7	School teacher	0.942	49	2008	Hülsheger et al., 2010; Kinman et al., 2011; Feuerhahn et al., 2013
#8	Non-traditional pre-searvice teacher	0.98	48	2014	Crosswell and Beutel, 2017; Brown et al., 2018; Mckay, 2019
#9	Emotional labor	0.981	42	2004	Näring et al., 2006; Çukur, 2009; Philipp and Schüpbach, 2010

Documents in both visualizations are qualitatively evaluated for identification of pivotal documents, areas of specialization, and research trends. In Figure 2, the cluster view provides an overview of TEL research between 1993 and 2020. The colder the color of the cluster (i.e., pink) is, the older the documents are. The warmer the color (i.e., orange), the newer the documents. In Figure 3, the timeline view adds additional insights by mapping the highly cited documents and the timing of new emerging topics. The evolution of TEL research is thus well-illustrated.

In view of the information provided in Table 7, Figures 2, 3, clusters with similar timelines were detected and grouped under four major themes after the documents were scrutinized. Specifically, Theme (1), the conceptualization of TEL is based on #1; Theme (2), the dimensional structuring and the explanatory theories and practices of TEL on # 9, #2, and #4; Theme (3), TEL-related teacher wellbeing on #7 and #3; and Theme (4), the factors linked to TEL on #0, #5, and #8. In the following section, these clusters will be analyzed as the intellectual basis, the themes will be reported, and the trends will be identified thereafter.

Theme (1), the conceptualization of TEL mainly draws upon documents in #1 teachers' perceptions (roughly from 1993 to 2003). Researchers in this period explored this theme through the lens of teachers' perceptions about their relationships with their students and colleagues or about their professions. In Hargreaves' (2000) study, TEL was investigated through teachers' perceived emotional closeness and/or distance with their students or “emotional geographies of schooling and human interaction” (p. 815). Specifically, elementary teachers experienced “psychic rewards” when they perceived close emotional bonds or emotional understanding with their students, while secondary teachers fended off and feared the emotions and felt a professional and physical distance from their students for fear of their possible interruptions to classroom management. Similarly, in Zembylas' (2003) case study, teachers' “contested professional discourse” (p. 318) revealed that the role of EL was positive as the science teacher perceived “gratifying emotional rewards in teaching” from her students' happiness in a supportive classroom learning community. In contrast, the teacher felt the negative impact of EL when she was dismissed by her colleagues for not adopting a teaching-to-the-test pedagogy. Looking through the lens of the profession, in Zembylas' (2005) study, teachers perceived themselves to be unprofessional if they showed “strong emotions” (p. 941) in discursive practices. In the same vein, in Winograd's (2003) study, teachers felt self-accusatory or self-blaming when they experienced the dysfunction of emotions.

As regards Theme (2), the dimensional structuring and the explanatory theories and practices of TEL are researched in # 9 emotional labor, #2 emotional capital, and #4 emotional practice (roughly from 2004 to 2007). In #9, studies reported on the dimensional structure of TEL. Besides the often-cited three-dimensional TELS originated from Diefendorff et al. (2005), the two-dimensional structure of deep acting and surface (Hülsheger et al., 2010; Philipp and Schüpbach, 2010) and the structure consisting of four dimensions (Näring et al., 2006; Çukur, 2009) were widely researched. Based on the TEL structure, researchers further explored TEL-related elements to build TEL conceptual models. They included the positive relationship between surface acting and emotional exhaustion (Näring et al., 2006), the reciprocal relationship between deep acting and health benefits over longer periods of time (Philipp and Schüpbach, 2010), a causal direction between surface and deep acting and their relationship with individual and organizational wellbeing (Hülsheger et al., 2010), and emotional consonance (one dimension of the TEL construct) and personal accomplishment.

In #2 and #4, researchers attempted to explain TEL through various theories and practices. According to the study by Zembylas (2007), both teachers' and students' emotional experiences are “forms of resistance to prevalent emotion norms” (p. 444) and are “profoundly influenced” (p. 459) by “emotional capital” (or termed as emotional resources), when it is systematically transformed into social and cultural capital. Zembylas (2003, p. 301) also proposed how teachers feel about EL in teaching can be reflected by their metaphorical language about emotions or “emotion metaphor.” In other words, the frustration and the joys that the science teacher expressed in science teaching revealed the negative and positive EL demanded in teaching. Yin and Lee (2012) interpreted TEL from emotional rules/feeling rules, which they believed can “direct teachers” EL (p. 58) when emotional labor was “not easily identified or recognized” (Zembylas, 2002, p. 201) under the cover of teachers' professional competence and ethical norms.

In cluster #4, various emotional practices of teachers were examined to account for TEL. In the context of new accountability and performance systems in educational reforms in the United Kingdom, teachers faced with capability procedures or with their capability challenged were required to shift from philanthropic emotion-giving to the prescriptive and formulaic emotion work, which was typical of their capable colleagues (Hebson et al., 2007), whereas under the marketization and modernization of the state education, in schools located in socially and economically deprived areas, teachers needed to fake or suppress emotions to develop positive learning environment and build rapport with pupils and parents to create a caring ethos as the selling feature of the school (Jenkins and Conley, 2007). In schools of children with special educational needs (SENs), teachers' emotional experiences were more like a rollercoaster, ranging from more positive and strong emotions (i.e., care and love) to darker emotions (i.e., anger, isolation, loneliness, and frustration) about the “expertism discourse” required by SEN (Mackenzie, 2012, p. 1079). Teachers being assessed or as the assessor under the practices of assessment in the educational system could go through intensely negative emotions, which accordingly limited their teaching effectiveness (Steinberg, 2008).

Theme (3), TEL-related teacher wellbeing draws on #6 personal welfare, #7 school teacher, and #3 extending conservation (roughly between 2008 and 2013). In #6, most studies found that surface acting strategy led to detrimental wellbeing with higher emotional exhaustion, depersonalization, lower personal accomplishment (Barber et al., 2011; Cheung et al., 2011; Kinman et al., 2011; Basim et al., 2013), and reduced job satisfaction (Cheung et al., 2011; Kinman et al., 2011), while deep acting had a mixed relationship with personal accomplishment (Kinman et al., 2011). Besides, emotional dissonance contributed to all three burnout dimensions (Cheung and Cheung, 2013). Interestingly, teachers with longer teaching experience were found subject to EL in Cheung and Cheung's (2013) study. However, Hosotani and Imai-Matsumura (2011) found that high-quality Japanese elementary school teachers, who excelled at classroom management and academic instruction, seemed to enjoy emotional wellbeing from “a virtuous cycle” where they were “well aware of their emotion management,” “executed emotional labor as educational professionals,” and were “willing to engage in emotion regulation without being affected by the negative aspects of emotional labor” (p. 1046).

To mitigate TEL-related ill wellbeing, various suggestions were found in the TEL literature under this theme. First, in terms of individual differences, extraverted teachers, in contrast to neurotic teachers, were less likely to experience emotional exhaustion (Basim et al., 2013). Emotional intelligence significantly moderated the impact of emotional job demands on teachers' surface acting and expression of naturally felt emotion (Yin, 2015). Second, resources can also function as moderating variables. Feuerhahn et al. (2013, p. 185) suggested that resources of emotional support and cognitive resources (i.e., teacher self-efficacy) could buffer the potentially detrimental impacts of the three emotional demands (i.e., parents' criticism, conflicts with colleagues, and emotional dissonance) on teachers. Similarly, conservation of resources (i.e., interpersonal influence) can buffer the negative effects of surface acting on reduced personal accomplishment (Park et al., 2014). Third, regarding the psychological aspects, teachers' psychological capital (PsyCap) (e.g., efficacy, optimism, hope, and resilience) (Cheung et al., 2011), teachers' implicit attitudes toward emotion regulation (Donker et al., 2020), and their enjoyment and frustration (Lee and Van Vlack, 2018) could mediate the relationship between TEL and the repercussions related to TEL. Fourth, burnout could also mediate the relationship between emotional dissonance and organizational citizenship behavior (Cheung and Cheung, 2013), and EL mediated the relationship between the teachers' perceptions of the school climate and emotional exhaustion (Yao et al., 2015).

In Theme (4), the factors linked to TEL (roughly starting from 2010 and to the end of 2020) and factors linked to in-service teachers' TEL draw on documents in #0 emotional experience and #5 predicting teacher while preservice teachers in #8 non-traditional preservice teacher. For in-service teachers, TEL-related factors were numerous. They can be psychological, socio-biographical, and classroom process ones. In terms of psychological factors, the use of cognitive reappraisal and more interpersonal agency in class was linked to reduced emotional exhaustion (Donker et al., 2020; Kang, 2020). While being adept at emotional control, teachers with active use of emotions and smiling service were likely to experience longer EL and more diverse and intense emotional states (Zhang et al., 2020b). Besides, teachers' perceptions of the school climate (Yao et al., 2015), their fantasy, personal distress (i.e., one aspect of empathy), perspective taking, and empathic concern (i.e., another aspect of empathy) (Huang et al., 2020), their emotional experiences and exhaustion (Keller et al., 2014), and their emotion regulation (Uzuntiryaki-Kondakci et al., 2020) could serve as predictors of EL. Interestingly, humor style was also found correlated with negative emotions (Liao et al., 2020). Teachers' discrete emotions were also related to TEL. For example, anger, as the predominant negative emotion felt in the classroom and a “multifaceted construct,” was positively related to surface acting and emotional exhaustion and negatively to job satisfaction and teachers' sense of efficacy (Burić and Frenzel, 2019; Taylor et al., 2020). Other emotions (i.e., positive ones: happiness, pride, enthusiasm, and liking; and negative ones: disliking and boredom) were also found to be linked to TEL (Taxer and Frenzel, 2015).

As regards socio-biographical factors, male teachers were found to be more likely to engage in surface acting than their female counterparts, and less senior teachers tend to display surface acting more frequently (Özdemir and Koçak, 2018; Olson et al., 2019; Donker et al., 2020). Besides, emotional job demands (i.e., social expectations and professional norms) (Yin et al., 2016), job characteristics (Huang et al., 2019), and the organizational cynicism level (Kuru Çetin, 2019) were found to relate to TEL. Koenig et al. (2018) even contended that professional development, even with its positive contribution to educators' professional knowledge and skills, could also evoke teachers' awareness of burnout, compassion fatigue, and self-care.

Particularly, classroom processes were also highlighted among others as a factor linked to teachers' EL (Burić and Frenzel, 2020). In their study, in classes with teachers who more frequently faked their emotions, their students displayed higher academic engagement, while teachers who often suppressed their feelings were perceived by their students as teachers who employed lower quality instructional strategies. Deep acting, by contrast, was unrelated to both teachers' instructional strategies and students' academic engagement.

However, the literature on preservice teachers' EL factors (in #8) is limited. Brown et al. (2018) found that their use of EL, particularly surface and deep acting, and their limited perceptions of emotional display rules were linked to the quality of their interactions with young children. To mitigate the negative effects of EL, identity development was suggested as a coping method for early-career teachers. For this purpose, teachers could build up their sense of self-care (Mckay, 2019) or demonstrate their agentic behaviors to navigate classroom experiences (Crosswell and Beutel, 2017).

**Figure 4:**
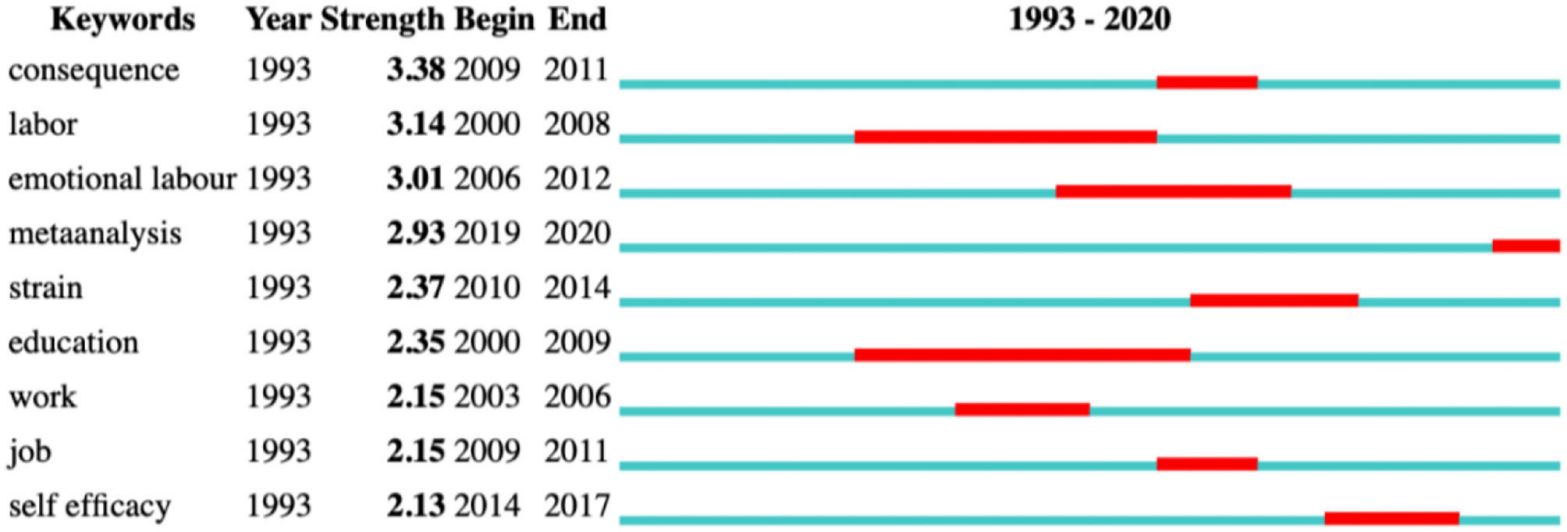
Top 9 keywords with the strongest citation bursts.

Furthermore, citation bursts above were provided as indicators of the most active areas of research (Figure 4) for they can identify the keywords that receive the most scholarly attention in a certain period of time (Zhou et al., 2019). Figure 4 lists the top nine keywords with bursts, which display the research hot issues and trends in TEL research. As is revealed in Figure 4, “consequence” and “labor” are the two strongest citation bursts, and “meta-analysis” represents the trend that attracts the latest scholarly interest. More importantly, Figure 4 can supplement and confirm the findings in Figures 2, 3.

The analysis above suggests that TEL research is both evolutionary and cumulative, which can be observed in three shifts. The first one is the shift of research focus from teachers' perception of TEL to context-related perspectives such as emotional rules, culture, and emotional sources to the impact of TEL on teacher wellbeing and teaching practices. The second shift is seen in theoretical implementation, where the researchers moved from a socio-cultural lens in the early period to a poststructuralist stance in recent years, with the former perspective on explaining how emotions are experienced through social relations and culture, and the latter explicating the critical positioning of power in the interpretation of EL. The third shift is reflected in research methods. TEL initially appeared in articles through descriptive and qualitative observations by early researchers in the 1990s (e.g., Hargreaves, 1998). Since the early 2000s, more systematic empirical research has been emerging; for instance, Näring et al. (2006) were among the first researchers in the field of education to adopt the emotional labor scale originally developed for organizational behavior research (see Brotheridge and Lee, 2003). Most recently, scholars have used the multimethod research approach embedding a series of independent studies within one single research project in response to the complexity of emotion (see Burić and Frenzel, 2019; Wang et al., 2020).

All in all, the past TEL research has enriched and well-complemented the EL research in the service sector. However, TEL may be significantly different from the EL in other human-service professions. The potentially significant difference requires that appropriate research focuses and research methods are adopted. On the one hand, because of the long duration of the service, episodes and chronic stressors (e.g., repeated experiences of unpleasant emotions) should become two interesting research focuses for future research endeavors; in light of the complexity of teaching contexts, subject-specific TEL and culture-specific TEL (e.g., Li and Liu, 2021) deserve future research attention. In contrast, as Dewaele and Wu (2021, p. 103) suggested, longitudinal designs may help “establish whether teachers' preferences change over time,” and that intervention studies and training may help teachers better regulate their emotions. Furthermore, as trait emotional intelligence (one personality trait) can predict TEL (Dewaele and Wu, 2021), it would be useful to examine to what extent other personality traits, such as L2 grit (Wei et al., 2020) and tolerance of ambiguity (Wei and Hu, 2019), have a similar influence. In a similar vein, it would be instrumental to investigate whether psychological individual differences [e.g., resilience, see Liu and Chu (2022)] other than personality traits may be linked to the TEL construct.

## Conclusion

The bibliometric analysis enables an objective perspective with statistics and co-citation network analysis provides key information on hotspots and interrelated trends of the research field (Guo et al., 2020). Both analyses contribute some empirical data to the evolution of TEL research. They reveal some interesting findings, which merit our particular attention. For example, in the case of the research area, linguistics ranks fourth (5.78%) in addition to the focal TEL research areas such as education (56.10%), psychology (32.37%), and wellbeing (7.50%). The place that linguistics holds signals more research attention from researchers in (applied) linguistics, indicating a potential to push the boundary of the knowledge of this topic. Indeed, as Prior (2019:518, emphasis in the original) rightly suggested, the study of emotion is “where L2 researchers and educators have a central role” and “any study of emotion must also be a study of *language*.”

Three major observations can be made based on the findings. First, the emergence of the research topic “TEL” coincides with the affective turn in the twenty-first century in some academic fields including management science, psychology, and L2 research. Second, the burgeoning TEL research indicates that education research has fended off the stereotyped priority over teachers' technical and intellectual knowledge to embrace the centrality of emotion and wellbeing in the teaching profession. Given this importance, more research on TEL is needed, especially in light of the much lower number (173) of TEL publications in WoS (*cf*. the number of EL publications, 3,546). Third, as previously mentioned, “any study of emotion must also be a study of *language*” (Prior, 2019, p. 518); the research on language teacher EL is part and parcel of the TEL research and is thus likely to inform emotion research in the neighboring fields and disciplines, such as education, psychology, sociology, and management science.

Despite a comprehensive picture of TEL publications that this article has managed to depict, two main limitations remain. First, the results of bibliometric analyses may be different when data sources other than the WoS are used (Mongeon and Paul-Hus, 2015; see Cansun and Arik, 2018). Second, due to the language constraint, an analysis combining national citation indexes (e.g., CNKI and Baidu Scholar based in China) may allow a more nuanced and contextualized understanding of TEL research. Future research may substantiate this study by adopting the suggestions mentioned above to pave for the common ground in interpreting TEL phenomena.

## Author Contributions

AW was responsible for the conceptualization, data collection, in-depth data analysis, original draft preparation, and funding. RW was responsible for the conceptualization, in-depth data analysis, original draft preparation, reviewing and editing, and supervision. Both authors contributed to the article and approved the submitted version.

## Funding

The writing of this paper was supported by the Educational Science Research Fund of Jiangsu Province (Grant No. D/2018/01/18).

## Conflict of Interest

The authors declare that the research was conducted in the absence of any commercial or financial relationships that could be construed as a potential conflict of interest.

## Publisher's Note

All claims expressed in this article are solely those of the authors and do not necessarily represent those of their affiliated organizations, or those of the publisher, the editors and the reviewers. Any product that may be evaluated in this article, or claim that may be made by its manufacturer, is not guaranteed or endorsed by the publisher.
